# Septic arthritis or adult-onset still’s disease: analyzing the causes of recurrent fever after arthroscopic combined anterior cruciate ligament and posterior cruciate ligament reconstruction: a case report

**DOI:** 10.1186/s12891-025-08938-9

**Published:** 2025-10-07

**Authors:** Zenan Tian, Jianlong Ni, Wang Wei, Qichun Song, Shanshan Liu, Dongjian Wang, Ruiying Li, Dazhi Wang, Zhihao Chen, Zhikun Jia, Jiajun Jiang, Zhibin Shi

**Affiliations:** 1 Department of Orthopaedic Surgery, The Second Affiliated Hospital Of Xi’an Jiaotong University, 157 Xiwu Road, Xi’an, Shaanxi Province China; 2 Department of Rheumatism and Immunology, The Second Affiliated Hospital Of Xi’an Jiaotong University, 157 Xiwu Road, Xi’an, Shaanxi Province China

**Keywords:** Recurrent fever, Septic arthritis, Adult-onset still's disease, AOSD, Ligament reconstruction, ACL, PCL, Combined ACL and PCL reconstruction, Case report

## Abstract

**Background:**

Adult-onset still’s disease and septic arthritis share similar symptoms—fever, joint pain, and limited mobility—making postoperative diagnosis difficult after combined anterior cruciate ligament and posterior cruciate ligament reconstruction. In this case, through a series of examinations and diagnostic treatment, the final diagnosis was adult-onset still's disease.

**Case presentation:**

A 41-year-old male developed recurrent fever and polyarthritis one week after undergoing arthroscopic combined anterior cruciate ligament and posterior cruciate ligament reconstruction. Clinical signs and tests were inconclusive. A multidisciplinary team was assembled to determine the diagnosis and establish a treatment plan. Despite broad-spectrum antibiotics, symptoms persisted. Typical adult-onset still's disease signs—rash, pharyngalgia, leukocytosis—emerged. Treatment with methylprednisolone, aspirin, and tocilizumab led to rapid improvement. Diagnosis was confirmed using Yamaguchi criteria. The patient was discharged in stable condition and remained symptom-free during follow-up. Knee function fully recovered.

**Discussion and conclusion:**

This may be the first reported case of adult-onset still's disease following combined anterior cruciate ligament and posterior cruciate ligament reconstruction. In conjunction with the relevant literature, we summarize the differences in clinical symptoms between septic arthritis and adult-onset still's disease. Reviewing the patient's hospitalization process, we discuss the "controversial" diagnostic and therapeutic measures taken by the multidisciplinary team, along with any doubts and considerations. The case highlights the diagnostic challenge of differentiating adult-onset still's disease from postoperative joint infection.

## Background

Fever is a common postoperative complication in surgical procedures. A study by Perlino CA found that noninfectious factors (including surgical trauma, transfusion reactions and medications) are the main cause of postoperative fever, accounting for 74%. Infectious factors (including wound infections, urinary tract infections and pulmonary infections) are secondary causes, accounting for 26% [1]. According to the time of fever onset, fever caused by drugs generally occurs within a few hours of drug administration and resolves after stopping the medication. Anesthesia-induced high fever typically occurs within 10 h after surgery, and timely administration of dantraline can effectively treat it. Fever resulting from surgical trauma appears within 48 h after surgery and can disappear spontaneously. Infections commonly cause fever within 3–5 days after surgery and may be accompanied by significant positive indicators, such as positive laboratory findings or cultures. Fevers caused by rheumatic immune diseases and tumors can persist for up to 3 weeks or even longer [1–4]. A fever with a temperature higher than 38.3℃ that occurs on several occasions and that lasts for at least 3 weeks and lacks a clear diagnosis after 1 week of hospital treatment is referred to as a fever of unknown origin (FUO) [5]. The common causes of FUO include infection, cancers, and noninfectious inflammatory diseases [5]. The patient experienced recurrent fever and polyarticular pain after 1 week of having arthroscopic combined anterior cruciate ligament (ACL) and posterior cruciate ligament (PCL) reconstruction, and the symptoms had been present for one week prior to admission. Based on the timeline of the symptoms and positive clinical signs, reasons such as trauma, anesthesia and drugs could be ruled out. There is a high possibility of preliminary suspicion of septic arthritis in joint infection and autoimmune diseases. However, it is necessary to rule out other infections, rheumatic immune diseases, tumors, hematological diseases, etc. [4, 6, 7].

Adult-onset still's disease (AOSD) is an agnogenic autoimmune inflammatory disease with typical symptoms, including recurrent fever, arthritis, rash and methemoglobinemia [8]. Laboratory tests typically show leukocytosis, increased neutrophil percentage, and elevated erythrocyte sedimentation rate (ESR) and C-reactive protein (CRP) levels [9]. AOSD arthritis is characterized by joint pain and limited mobility, with the knee, wrist and ankle joints being more susceptible to being affected [10, 11]. AOSD diagnosis is based on exclusion, as there are no specific serological or histopathological markers [12]. Several diagnostic criteria exist for AOSD, among which the Yamaguchi criterion is the most widely cited, with a sensitivity of 93.5% [13–15].

The typical clinical manifestations of septic arthritis include fever, joint pain, limited mobility, and erythema. Laboratory findings show leukocytosis and elevated ESR and CRP levels [16]. According to the guidelines for the management of septic arthritis in native joints published by the European Bone and Joint Infection Society (EBJIS) in 2022, septic arthritis should be kept in mind in any patient with a painful and/or inflamed joint (redness, hot, swelling, synovial effusion, and/or purulent drainage) with or without a fever. No clinical parameters can exclude or confirm septic arthritis [6]. Given the overlapping symptoms between septic arthritis and AOSD—including fever, joint pain, and elevated inflammatory markers—this case highlights a rare but clinically significant diagnostic dilemma. This report contributes to the existing literature by describing a novel postoperative presentation of AOSD, offering insight into the differential diagnosis of postoperative fever in patients after ACL, PCL or combined ACL and PCL reconstruction.

## Case presentation

### Stage 1: from symptoms onset to hospitalization

A 41-year-old Chinese male underwent arthroscopic combined ACL and PCL reconstruction following a traumatic knee injury. One week after surgery, he developed recurrent fever (up to 38.5 °C), chills, and polyarthritis. He self-administered antibiotics and etoricoxib, which briefly reduced symptoms, but high fever recurred within three days.

At admission, his temperature was 39.5 °C. The left knee showed only mild swelling, negative floating patella sign, and limited flexion to 90°. Chest CT showed chronic pulmonary changes and mild pleural effusion. Laboratory tests (Table 1 on admission) revealed leukocytosis (WBC 24.64 × 10⁹/L), neutrophilia (86.5%), elevated CRP (165.04 mg/L) and ESR (96 mm/h), and mildly abnormal liver function. ANA was weakly positive; RF was negative.

**Table 1:** Laboratory examination results during hospitalization

Component	On admission	1 st MDT	2nd MDT	Reexamination	Reference range
WBC (× 10^9^/L)	24.64	22.93	22.08	41.88	3.50–9.50
Neutrophils (%)	86.5	90.7	86.7	83.90	40–75
Hemoglobin (g/L)	113	89	116	110	130–175
Platelet (× 10^9^/L)	456	539	522	523	125–350
CRP (mg/L)	165.04	125.26	< 10	-	< 10
ESR (mm/h)	96	116	21	-	0–15
Total protein (g/L)	56.5	64.5	52.8	66.1	60–85
Albumin (g/L)	28.8	31.6	29.7	33.3	35–55
bilirubin, direct (umol/L)	1.20	4.65	2.46	1.62	< 4
bilirubin, total (umol/L)	4.20	13.90	11.10	7.60	< 23
ALT (U/L)	190.80	252	94	115	9–50
AST (U/L)	92.50	115	23	54	15–40
ALP (U/L)	162	288	166	108	45–125
INR	1.12	1.11	0.93	-	0.9–1.3
PT (s)	12.7	12.6	10.3	-	9.8–12.1
APTT (s)	25.0	19.5	17.8	-	22.7–31.8
D-Dimer (ng/mL)	1520.00	1050.00	700.00	-	0–1000
ANA	weakly positive	-	-	-	negative
RF (IU/L)	< 11.60	-	-	-	0–15.9
IL-6 (pg/mL)	74.98	198.35	-	-	0–5.4
IL-1β (pg/mL)	15.38	-	-	-	0–12.4

*ALP* Alkaline phosphatase, *ALT* Alanine amino transaminase, *APTT* Activated partial thromboplastin time, *ANA* Antinuclear antibody, *AST* Aspartate aminotransferase,*CRP* C-reactive protein, *ESR* Erythrocyte sedimentation rate, *INR* International normalized ratio, *PT* Prothrombin time, *RF* Rheumatoid factor, *WBC *White blood cell

Combining the patient's symptoms and history of combined ACL and PCL reconstruction, we initially suspect it to be a postoperative joint infection.

### Stage 2: days 1–12 of admission

Upon admission, the patient tested negative for COVID-19 and urine cultures. Bone marrow aspiration revealed peripheral leukocytosis. Five sets of aerobic and anaerobic blood cultures were performed during febrile spikes—all were negative. A multidisciplinary team (orthopedics, infectious disease, rheumatology, hematology, respiratory medicine, pharmacy) was formed. Initial suspicion included sepsis and septic arthritis; empirical ceftriaxone was administered. Viral serologies (TORCH, EBV) and repeated COVID-19 testing were negative.

On day 3, the patient’s fever rose to 40 °C, accompanied by a punctate rash on the neck and sore throat. Imaging revealed soft tissue edema and joint effusion in the left knee. Meropenem was initiated for 6 days without clinical improvement; later replaced by moxifloxacin, cefotiam, and linezolid. Despite broad-spectrum antibiotics, the fever persisted. Fungal markers and TB screening were negative. With poor response to antibiotics and atypical features, the MDT began to suspect AOSD.

### Stage 3: diagnostic clarification and treatment

On day 13, a case discussion concluded that septic arthritis could be ruled out. AOSD was strongly suspected. Further testing (Brucella agglutination, PPD, NGS, bone marrow) excluded infection and malignancy. NGS showed parvovirus, likely contamination. The patient received diagnostic treatment with methylprednisolone, aspirin, and tocilizumab—leading to rapid improvement of fever and rash.

**Fig. 1:**
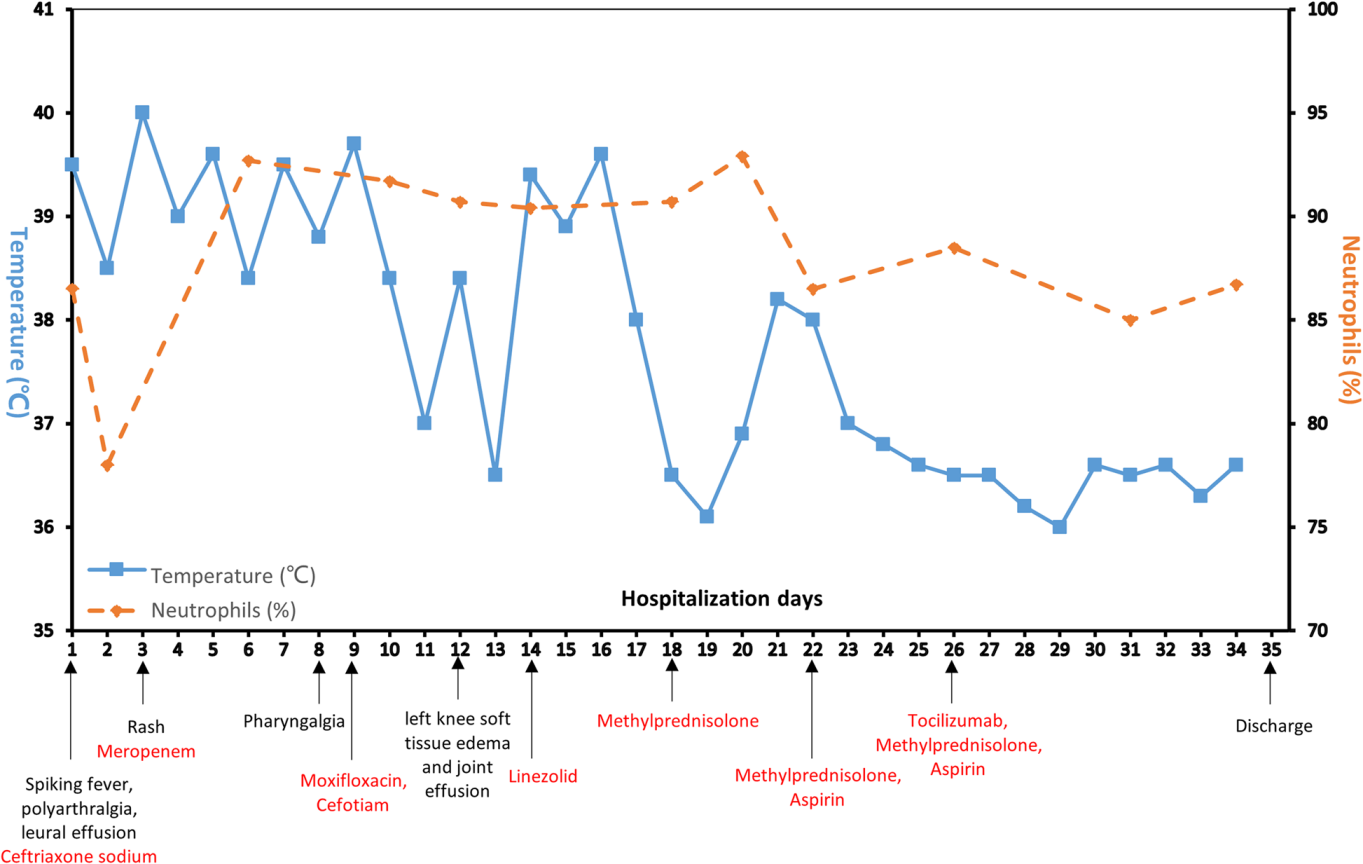
Body temperature, neutrophils, important symptoms, and drugs timeline during hospitalization

A second discussion on day 34 confirmed AOSD per Yamaguchi criteria (four major: spiking fever, arthralgia, rash, leukocytosis; two minor: sore throat, negative ANA/RF). Inflammatory markers normalized (CRP < 10 mg/L; ESR 21 mm/h). The patient was discharged with knee range of motion 0–85° and no active symptoms. A brief diagram of the patient’s disease changes is shown in Fig. 1.

### Stage 4: follow-up

**Fig. 2:**
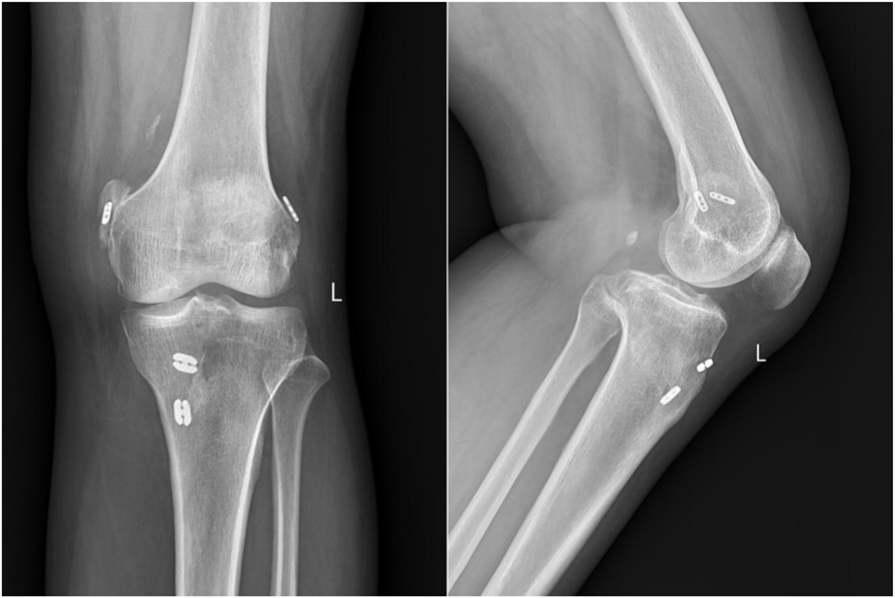
Radiographs of the knee in adult-onset still's disease. Postoperative changes of the combined ACL and PCL reconstruction with good internal fixation position

**Fig. 3:**
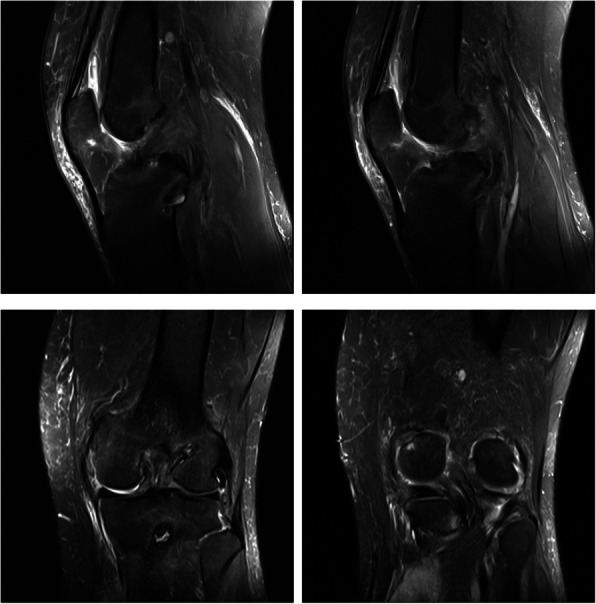
MRI of the knee in adult-onset Still’s disease. Postoperative changes of the combined ACL and PCL reconstruction with good internal fixation position and signal. Small amount of fluid accumulation in the joint and slight swelling of the surrounding soft tissues

Post-discharge, the patient continued prednisone (50 mg/day) and wore a knee brace. At two-week and one-month follow-ups, symptoms remained controlled. Labs were stable. X-ray and MRI showed good internal fixation and mild joint effusion (Figs. 2 and 3). By three months, joint mobility was normal with no symptom recurrence.

## Discussion

Since Bywater first proposed the concept of AOSD in 1971, the condition has been extensively characterized in the literature [17]. However, we found no prior reports of AOSD manifesting shortly after arthroscopic combined ACL and PCL reconstruction despite comprehensive searches across multiple databases (PubMed, EMBASE, Cochrane). This report, therefore, adds novel clinical insight by highlighting a rare postoperative presentation of AOSD that closely mimics septic arthritis. It emphasizes the need for heightened diagnostic vigilance when evaluating postoperative fever, especially when conventional treatments for infection prove ineffective. The purpose of this case report is not to dramatize the diagnostic process, but to document the real-world complexity of clinical decision-making under diagnostic uncertainty. The multidisciplinary approach adopted in this case—although at times appearing iterative—reflects the evolving nature of differential diagnosis in ambiguous postoperative presentations. This detailed presentation may assist clinicians in recognizing subtle clinical features suggestive of AOSD in similar scenarios and encourage a broader differential diagnosis when infection is not substantiated by microbiological evidence.

Based on this case and relevant literature, we summarized the differences in clinical symptoms between septic arthritis and AOSD (Table 2) [16, 18–20].

**Table 2:** Key clinical differences between septic arthritis and adult-onset Still's disease (AOSD) in the context of postoperative fever—emphasizing diagnostic pitfalls and distinguishing features

Clinical features	Septic arthritis	AOSD
Fever	Most cases are mild and only 30%–40% of individuals have a temperature > 39℃	Peak fever and the highest temperatures (> 39 ℃) occurs at night
Arthralgia and arthritis	Most cases are monoarticular and only 10%−20% of individuals have a polyarticular disease	Most cases are polyarthritis
Dermatologic manifestations	Erythema around the affected joint	A macular or maculopapular evanescent salmon-pink skin rash appears together with the fever spikes
Pharyngalgia	Generally, not present, but can occur when accompanied by throat infections	It occurs before or during the first month of each disease flare
Others	Occur rarely but shivering can occur	It is common and includes myalgia, enlargement of the lymph nodes, splenomegaly, hepatomegaly, pleurisy, pericarditis, weight loss, etc

### Experiences

Looking back at the entire diagnosis and treatment process, the multidisciplinary team took some"controversial"measures:

1. Multiple studies, such as the clinical guidelines published by EBJIS in 2022, have emphasized that joint fluid aspiration and culture are essential for the diagnosis of septic arthritis [6, 16, 21]. Why was joint fluid aspiration and culture not performed in this case?
  The blood cultures were repeatedly negative, indicating a lack of positive evidence for infection. The patient did not exhibit significant symptoms in the operated joint, and the floating patella test was negative. Additionally, postoperative puncture may lead to secondary infection. Through the search of relevant literature, we found a case where AOSD was misdiagnosed as a prosthetic joint infection (PJI) and another case where AOSD was misdiagnosed as septic arthritis. Both of the patients of these cases underwent joint fluid aspiration and culture, and the results suggested joint infection [22, 23]. Based on the two cases, the decision was correct to some extent in not performing joint fluid aspiration and culture.
2. EBJIS suggests that arthroscopic debridement should be performed as soon as the suspicion of septic arthritis is raised in cases with acute symptoms or in the early postoperative setting, even if the microbiological results are still pending [6]. Additionally, multiple studies have indicated that arthroscopic debridement is highly effective in septic arthritis treatment, especially after ACL reconstruction [6, 16, 21]. So why was arthroscopic debridement not performed in this case?
  We observed that the patient had polyarticular pain, but the operated joint did not exhibit significant swelling or pain. Arthroscopic debridement is an invasive treatment that increases the risk of nosocomial infection and would incur additional costs of treatment to the patient. Therefore, instead of arthroscopic debridement, we treated the patient with powerful and empirical antibiotics.
3. The patient's symptoms and examinations indicated inflammation on admission, and we considered the possibility of infection. Why was septic arthritis suspected instead of other related infections?
  On the one hand, EBJIS states that a high suspicion of septic arthritis should be kept in mind in any patient with a painful and/or inflamed joint with or without a fever. On the other hand, multiple studies have found that septic arthritis is more common in infections after ACL reconstruction. Furthermore, arthroscopic combined ACL and PCL reconstruction involves a longer surgical duration and greater surgical trauma than isolated ACL reconstruction or PCL reconstruction, which increases the risk of postoperative infection [6, 24–26]. Therefore, septic arthritis is given priority consideration in this case. However, we acknowledge that omitting joint aspiration in this case may be viewed as a limitation. According to EBJIS, joint fluid aspiration and culture are considered essential for diagnosing septic arthritis, particularly in the early postoperative period. Although our patient exhibited no overt signs of joint infection—such as erythema, significant swelling, joint line tenderness, or floating patella—the absence of aspiration removes a valuable source of diagnostic clarity. The decision was influenced by the patient’s mild local signs, concerns about secondary infection from invasive procedures, and multiple negative blood cultures. Nonetheless, we recognize that many institutions would perform aspiration as part of good clinical practice, and future similar cases may benefit from including this step even in the absence of classical features.

### Concerns and reflection

Furthermore, there is controversy over the diagnostic criteria for septic arthritis because of the lack of high-quality evidence-based research, which also increases the difficulty of diagnosis in this case [16]. First, we considered septic arthritis as a strong possibility but could not exclude AOSD. Subsequent examinations, including five blood cultures performed during the periods of elevated body temperature, did not reveal evidence of infection. Empiric antibiotic treatment was ineffective. Septic arthritis was excluded at 13 days after admission and lasted for 13 days. Further examinations ruled out infection, blood disorders and tumors. In combination with effective diagnostic treatment, the diagnosis of AOSD was confirmed on the 34th day of admission, after a total of 31 days. Looking back at the entire diagnosis and treatment process, the multidisciplinary team presents the concerns and reflections:

1. Within the 13 days from suspicion to exclusion of septic arthritis, we performed five blood cultures (aerobic and anaerobic) during the periods of elevated body temperature, and all results were negative. Regarding the number of blood cultures, the guidelines by EBJIS in 2022 suggest that at least two sets of aerobic and anaerobic blood cultures should be performed, but they do not provide a specific upper limit [6]. However, the patient had taken antibiotics before admission, which necessitates an increased number of blood cultures to eliminate the impact of antibiotics. It is worth considering whether five blood cultures were excessive for the diagnosis? Could the number be reduced to 3-4? We should summarize our experience from this case and our clinical practice.
2. Referring to the two cases of AOSD, joint fluid aspiration and culture were performed, and PJI and septic arthritis were suspected according to the results. Arthroscopic debridement was performed several times in these two cases [22, 23]. Therefore, it remains a matter of clinical judgment whether joint fluid aspiration and culture should be universally performed in cases where septic arthritis and AOSD are both in the differential diagnosis. In retrospect, we acknowledge that not performing aspiration may be considered a flaw in the diagnostic pathway. While our decision was based on the absence of typical local joint infection signs and the concern for iatrogenic contamination postoperatively, we recognize that joint aspiration remains a widely endorsed step in best clinical practice. As such, we have added this reflection as a limitation in the discussion section to better inform future cases.
3. On the 14th day of admission, the patient had been experiencing fever with a temperature higher than 38.3℃ on several occasions that lasted for at least 3 weeks and lacked a clear diagnosis after 1 week of hospital treatment. According to the criteria for FUO, the patient could then be diagnosed with FUO [5]. The common causes of FUO are infection, tumors and noninfectious inflammatory diseases. AOSD is the most common noninfectious inflammatory disease [5]. If diagnosed as FUO, AOSD can be considered and confirmed after excluding infection (multiple negative blood cultures, negative bacterial, fungal and viral tests) and tumor (ruled out by bone marrow aspiration and NGS). Comparing the 31 days it took to diagnose AOSD in this case, diagnosing AOSD based on the FUO criteria could significantly reduce the time needed for confirmation.

## Conclusion

In conclusion, AOSD and septic arthritis share similar clinical symptoms. It is challenging to make a definitive diagnosis because the positive evidence is insufficient, which may prolong the diagnosis and treatment time, as seen in this case. In such situations, we believe that setting up a multidisciplinary team is beneficial for diagnosis and treatment. To shorten the diagnosis time and avoid unnecessary diagnosis and treatment, we should pay attention to subtle differences between symptoms and examinations and adopt empirical and diagnostic treatment measures.

## Data Availability

No datasets were generated or analysed during the current study.

## Abbreviations

AOSD: Adult-onset Still's disease
ACL: Anterior cruciate ligament
PCL: Posterior cruciate ligament
FUO: Fever of unknown origin
ESR: Erythrocyte sedimentation rate
CRP: C-reactive protein
EBJIS: European Bone and Joint Infection Society
EBV: Epstein-Barr virus
NGS: Next generation sequencing
PJI: Prosthetic joint infection
